# Real-world assessment of the effectiveness of posaconazole for the prophylaxis and treatment of invasive fungal infections in hematological patients: A retrospective observational study

**DOI:** 10.1097/MD.0000000000026772

**Published:** 2021-07-30

**Authors:** Xiaochen Chen, Jianxiang Wang, Sanbin Wang, Jie Jin, Junmin Li, Sujun Gao, Jianyong Li, Juan Li, Qifa Liu, Yu Hu, Dongjun Lin, Zimin Sun, Jianmin Yang, Jianda Hu, Xiaoxiong Wu, Xiaojun Huang, Zonghong Shao, Qi Deng, Chun Wang, Li Liu, Hu Chen, Jingbo Wang, Xudong Wei, Jianping Shen, Xi Zhang, Depei Wu

**Affiliations:** a Department of Hematology, The First Affiliated Hospital of Soochow University, Suzhou, Jiangsu Province, China; b Leukemia Diagnosis and Treatment Center, Blood Diseases Hospital, Chinese Academy of Medical Sciences, Tianjin, China; c Department of Hematology, Kunming General Hospital, Chengdu Military Region, Kunming, Yunnan Province, China; d Department of Hematology, The First Affiliated Hospital of Medical School of Zhejiang University, Hangzhou, Zhejiang Province, China; e Department of Hematology, Ruijin Hospital Affiliated to Medical College of Shanghai Jiaotong University, Shanghai, China; f Department of Hematology, The First Bethune Hospital of Jilin University, Changchun, Jilin Province, China; g Department of Hematology, The First Affiliated Hospital of Nanjing Medical University, Nanjing, Jiangsu Province, China; h Department of Hematology, The First Affiliated Hospital, Sun Yat-Sen University, Guangzhou, Guangdong Province, China; i Department of Hematology, Nanfang Hospital Affiliated to Southern Medical University, Guangzhou, Guangdong Province, China; j Department of Hematology, Wuhan Union Hospital Affiliated to Tongji Medical College, Huazhong University of Science and Technology, Wuhan, Hubei Province, China; k Department of Hematology, The Third Affiliated Hospital, Sun Yat-Sen University, Guangzhou, Guangdong Province, China; l Department of Hematology, Anhui Provincial Hospital, The First Affiliated Hospital of USTC, Hefei, Anhui Province, China; m Department of Hematology, Changhai Hospital, Shanghai, China; n Department of Hematology, Fujian Medical University Union Hospital, Fuzhou, Fujian Province, China; o Department of Hematology, Fourth Medical Center of PLA General Hospital, Beijing, China; p Department of Hematology, Peking University People's Hospital, Beijing, China; q Department of Hematology, Tianjin Medical University General Hospital, Tianjin, China; r Department of Hematology, Tianjin First Central Hospital, Tianjin, China; s Department of Hematology, Shanghai General Hospital, Shanghai, China; t Department of Hematology, Tangdu Hospital, Xi’an, Shaanxi Province, China; u Department of Hematopoietic Stem Cell Transplantation, 307 Hospital of PLA, Beijing, China; v Department of Hematology, Aerospace Central Hospital, Beijing, China; w Department of Hematology, Henan Cancer Hospital, Zhengzhou, Henan Province, China; x Department of Hematology, Zhejiang Provincial Hospital of TCM, Hangzhou. Zhejiang Province, China; y Department of Hematology, Xinqiao Hospital, Chongqing, China.

**Keywords:** hematological malignancy, hematopoietic stem cell transplantation, invasive fungal disease, posaconazole, prophylaxis

## Abstract

The aim of the study was to analyze the efficacy of posaconazole for the prophylaxis and treatment of invasive fungal diseases (IFDs) in patients with hematological malignancies.

In this retrospective observational multi-center study, 762 patients from 25 Chinese hematological centers were enrolled. Inclusion criteria were patients with hematological malignancy or they had undergone hematopoietic stem cell transplantation and received at least 1 dose of posaconazole. The primary endpoints were the observation of breakthrough rates and the clinical efficacy of posaconazole prophylaxis. The secondary endpoint was the efficacy of posaconazole for the treatment of IFDs.

Of the 762 enrolled patients, 456 (59.8%) were prescribed posaconazole prophylactically while 243 (31.9%) received posaconazole as an IFD treatment (12 proven, 61 probable, 109 possible, and 61 unclassified IFD cases) for ≥7 days. The overall IFD breakthrough rate (probable cases) for the ≥4 days prophylactic treatment (n = 445) group was 1.6% (95% Cl: 0.6%–3.2%), with breakthrough rates of 2.6% for acute myeloid leukemia/myelodysplastic syndrome patients undergoing chemotherapy and 2.2% for hematopoietic stem cell transplantation patients. For primary antifungal prophylaxis, the breakthrough rate was 1.9% and for secondary antifungal prophylaxis 0%. The overall effective IFD remission rate of patients treated for ≥7 days with posaconazole was 56.0% and the effective remission rate of proven/probable/possible IFD cases was 59.3%. The effective remission rate of posaconazole as salvage therapy was 50% (95% CI: 32.4%–67.6%) including 75% (CI: 19.4%–99.4%) for *Aspergillus* infections.

The present retrospective study confirmed posaconazole as IFD prophylaxis and medication for hematological malignancy patients undergoing various treatments in China.

## 1 Introduction

Over the past 20 years or so, invasive fungal diseases (IFDs) have become one of the main causes of death in hematological patients treated with high-intensity chemotherapy and/or a hematopoietic stem cell transplantation (HSCT).^[1,2]^ Neutrophils are of particular importance for immune defense against fungal infections. They makeup about 50% to 70% of all blood cells and carry out the first innate immune response at a site of infection. Granulocyte colony-stimulating factor, which is the main factor for proliferation and differentiation of myeloid progenitor cells into neutrophils, is the ligand for the granulocyte colony-stimulating factor receptor, which is encoded by the colony-stimulating factor 3 receptor (*CSF3R*) gene. Mutations of the *CSF3R* gene are associated with chronic neutrophilic leukemia, acute myeloid leukemia (AML), atypical chronic myelogenous leukemia, as well as severe congenital neutropenia and myelodysplastic syndrome (MDS).^[3]^ Neutrophil responses to fungal pathogens include the release of reactive oxygen species and phagocytosis, and the secretion of neutrophil extracellular traps, which comprise chromatin threads with attached antimicrobial components from granules and cytoplasm that trap and entangle fungal pathogens after binding to their anionic surfaces.^[4]^ IFD recognition is difficult due to the low accuracy of early clinical diagnosis^[5]^ and because it takes 3 to 5 days for culture test evidence to become available. Therefore, empirical and prophylactic therapies are frequently given to patients who are suspected of having a fungal infection or have been clinically diagnosed with reduced neutrophil numbers and for patients who have undergone transplant surgery.

In China's invasive fungal infections research study, it was shown that antifungal therapy could markedly improve the curative effect in proven, probable/possible, and suspected IFD patients, and that prophylactic therapy reduced the incidence of IFD.^[6]^ In addition, randomized controlled trials have shown that prophylaxis with fluconazole can significantly reduce *Candida* infections and decrease the mortality of *Candidiasis*-infected patients, which thus verified the rationality of antifungal prophylactic therapy.^[7–9]^ Posaconazole is a drug with an extended-spectrum antifungal activity and is more effective in preventing IFDs and reducing the risk of IFD breakthrough than fluconazole or itraconazole.^[10]^ As 1 of the second generation of triazole drugs, posaconazole has good toxicology characteristics^[11]^ and high antifungal activity against new-type resistant pathogen strains. A real-world study in Germany found that antifungal posaconazole salvage therapy was effective in 72.2% of aggressive *Aspergillosis* cases after voriconazole treatment had failed.^[12]^ In addition, previous studies reported that prophylactic posaconazole therapy was effective in preventing IFDs,^[13–16]^ though others have reported that certain IFD breakthroughs occurred despite prophylactic posaconazole treatment, which were attributed to a shift to non-*Aspergillus* spp and the low doses of posaconazole administered.^[17,18]^

In China, posaconazole has been in use since 2014 and the present study aimed to analyze retrospectively its clinical effects with the breakthrough point of prophylaxis and treatments, as well as salvage therapy in hematological patients who had been treated with posaconazole from June 30, 2014 to October 31, 2015. There is a particular focus on the risk factors for IFD breakthrough after prophylactic posaconazole treatment.

## 2 Methods

The ethics committee of the First Affiliated Hospital of the Soochow University approved the multi-center, retrospective, single-arm study (number: 2015-046) and written informed consent was not required.

**Figure 1:**
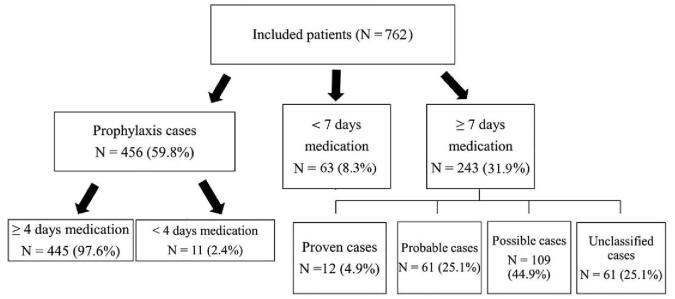
Flow chart of the present study.

A total of 762 eligible cases were analyzed in 25 Chinese hematological centers (Figure 1, Supplemental Digital Content). The research physicians reviewed the hospital electronic medical records database from the discharge time-point of October 31, 2015 back to June 30, 2014; eligible cases according to the inclusion criteria were continuously screened out. Each center was allowed to enroll ≤50 cases and all cases were traced back to June 30, 2014.

*Inclusion criteria*: Patients were retrospectively included in the study if they had been hospitalized to receive treatments for hematopathy or HSCTs and discharged before a specified date. These patients received at least 1 dose of posaconazole prophylactically or as a treatment for IFDs from June 30, 2014 to October 31, 2015.

*Exclusion criteria*: Patients who had previously participated in interventional clinical trials of other antifungal agents during hospitalization.

Combined use of permitted drugs together with combinations of drugs prohibited in the study are detailed in the instructions for posaconazole administration.

### 2.1 Medication

Prophylactic posaconazole monotherapy is the main treatment used in clinical practice, with a median duration of 14 days; 13 days prophylaxis for patients receiving chemotherapy; and 19 days for transplant patients. Posaconazole medication was initiated before chemotherapy and after transplantation. However, because this was a retrospective study, the dosage of posaconazole was determined according to the manufacturer's instructions and the clinicians’ prescriptions. The non-initial prophylactics and treatments, as well as the combination drugs administered, were fluconazole, voriconazole, caspofungin, micafungin, and/or itraconazole.

### 2.2 Endpoints of the study

#### 2.2.1 Primary endpoint

The breakthrough rate of IFD and the proportion of patients switching to systemic antifungal therapy for the evaluation of the clinical efficacy of posaconazole prophylaxis in patients with IFDs.

#### 2.2.2 Secondary endpoints

The efficacy of posaconazole for the treatment of IFD, usage of posaconazole in patients with hematologic disease in China, characteristics of patients receiving posaconazole for IFD prevention or the treatment drug and analysis of the risk factors for IFD breakthrough during posaconazole prophylaxis.

#### 2.2.3 Exploratory research endpoint

Evaluation of the clinical effectiveness of posaconazole as salvage therapy for IFD.

### 2.3 Objective indicators

#### 2.3.1 Posaconazole as a prophylactic drug

The definition of antifungal prophylaxis refers to patients with high-risk factors for IFD and antifungal drug used for the prevention of IFD before the patients develop clinical symptoms.

The primary objective was the breakthrough rate of IFD. The secondary objective was the proportion of patients who have initially prescribed posaconazole as prophylaxis therapy and who then converted to empirical or pre-emptive systemic antifungal treatment.

*Diagnosis of IFD and IFD breakthrough*: defined according to the European Organization for Research and Treatment of Cancer/Invasive Fungal Infections Cooperative Group and the National Institute of Allergy and Infectious Diseases Mycoses Study Group (EORTC/MSG) guidelines as proven, probable and possible cases,^[19]^ and unclassified cases according to the classification of Maertens et al^[20]^ The definition of IFD breakthrough was defined as proven/probable IFDs, based on the EORTC/MSG standard, which occurred between ≥4 days after posaconazole prophylaxis initiation and ≤7 days after discontinuation of prophylaxis therapy.

The IFD breakthrough rate = (proven diagnosed patients)/(patients with continuous use of drug ≥4 days) × 100%.

If the physician's diagnosis of IFD did not conform to the EORTC/MSG criteria, all IFD diagnoses were based on the data recorded in the case report form.

#### 2.3.2 Use of posaconazole as antifungal therapy

The primary objective was the remission rate including complete remission or partial remission of proven/probable diagnosed IFD patients who received posaconazole as a salvage therapy drug at the end of posaconazole treatment. The secondary objectives were the effective remission rate for proven/probable/possible IFD after posaconazole therapy (≥7 days) at the end of posaconazole treatment and the current usage status of posaconazole as a treatment for IFD.

Exploratory objectives were the proportion of patients with complete remission/partial remission/stable disease at the end of posaconazole treatment and the effective remission rates at the end of posaconazole therapy (posaconazole usage ≥7 days), as well as the effective remission rates of posaconazole treatment as salvage therapy.

The definition of salvage therapy had to meet 4 conditions: (1) proven diagnosed IFD; (2) posaconazole as a non-initial treatment drug; (3) the duration of antifungal treatment before posaconazole was >7 days; and (4) the reason that antifungal treatment was switched to posaconazole was adverse events or poor efficacy of the initially prescribed drug(s).

### 2.4 Sample size

Since this was a descriptive study, there was no pre-test of the hypothesis; the sample size was calculated based on the expected margin of error according to the estimated IFD breakthrough. Recently, the results of the China's invasive fungal infections research initiative have indicated that the incidence rate of proven or clinically diagnosed IFD in patients who received prophylaxis treatment was about 7% in those undergoing HSCT and 3% in the MDS/AML patient group (before posaconazole was available in China). If the confidence level is 0.05, the confidence interval is 95% (margin of error ±2.5%), and if the incidence of IFD can be assumed to decrease by at least 50% after posaconazole prophylaxis, then the expected breakthrough will be 2.0% to 3.5% or lower. As shown in Table 1, Supplemental Digital Content, when the incidence of IFD was 4%, the sample size of the prevention group was 236 patients.

In addition, considering that the breakthrough in the calculation process was a stratified analysis on different populations groups (HSCT and chemotherapy), even posaconazole could be used as the therapeutic drug. Therefore, we prospected the ratio of patients undergoing chemotherapy and transplants as 1:1 and the ratio of patients receiving prophylaxis and treatment as 2:1. Thus, the sample size of about 800 cases was sufficient and the number of patients receiving prophylaxis in the chemotherapy group was ≥236 cases.

### 2.5 Statistical analysis

All analysis was carried out using SPSS software version 17 (SPSS Chicago, IL, US).

For quantitative variables, the descriptive statistics of the index are shown as the number of cases (missing number), arithmetic mean, standard deviation, median, minimum, and maximum values. For qualitative categorical variables, the frequency and percentage of corresponding variable values are given. The categorical endpoints and 95% CI were used to estimate the clinical effect of posaconazole, including the IFD breakthrough, the proportion of prophylaxis treatment failures and the proportion of systemic antifungal treatments.

## 3 Results

### 3.1 Demographic information of the patients

A total of 762 patients were screened and enrolled of whom 456 were treated prophylactically with posaconazole accounting for 243 (59.8%) patients who received posaconazole treatment therapy for ≥7 days. Of these, 12 were proven, 61 probable, 109 possible, and 61 cases of unclassified IFDs (Fig. 1).

Further comparison of the patients who were given posaconazole as prophylaxis treatment or as therapy showed that the majority had an Eastern Cooperative Oncology Group score of 1 and that there were no statistically significant differences between the 2 groups regarding comorbidities (Table 1). Hematological malignancy patients accounted for the majority of those in the 2 groups (91.2% and 90.1%), with AML being diagnosed most frequently (54.8% and 46.6%) followed by acute lymphocytic leukemia (ALL) (20.7% and 26.9%).

**Table 1:** The basic characteristics of enrolled patients treated with posaconazole as prophylaxis or therapy medication.

		Prophylaxis N = 456	Therapy (≥7 days) N = 243
Age (year)	Number	456	243
	Mean (SD)	37.4 (16.5)	37.7 (17.0)
Gender, n (%)	Male	254 (55.7)	154 (63.4)
	Female	202 (44.3)	89 (36.6)
Weight (kg)	Mean (SD)	60.9 (14.1)	58.9 (12.7)
ECOG score, n (%)	0	99 (21.7)	56 (23.0)
	1	195 (42.8)	86 (35.4)
	2	103 (22.6)	54 (22.2)
	3	51 (11.2)	38 (15.6)
	4	8 (1.8)	9 (3.7)
	5	0	0
ANC/mm^3^, n (%)			
ANC ≥1000	ANC ≥1500	184 (40.4)	149 (61.3)
	ANC 1000–1500	44 (9.6)	
ANC 500–1000		47 (10.3)	30 (12.3)
ANC <500	ANC 100–500	85 (18.6)	64 (26.3)
	ANC <100	75 (16.4)	
Not measured		21 (4.6)	0
Comorbidities, n (%)	Diabetes	25 (5.5)	13 (5.3)
	Chronic hepatitis	26 (5.7)	19 (7.8)
	Gastroesophageal disease	7 (1.5)	5 (2.1)
	Acquired immunodeficiency	0	3 (1.2)
	Hereditary immunodeficiency	2 (0.4)	0
	Autoimmune disease	5 (1.1)	2 (0.8)
Adverse events of posaconazole, n (%)	Diarrhea	40 (8.8)	17 (7.0)
	Vomit	35 (7.7)	12 (4.9)
Diagnosis of primary hematologic diseases			
Diagnoses of the full analysis set, n (%)	Hematological malignancies	416 (91.2)	219 (90.1)
	Missing	0	0
Type of hematological malignancies, n (%)	ALL	86 (20.7)	59 (26.9)
	AML	228 (54.8)	102 (46.6)
	CLL	2 (0.5)	0
	CML	7 (1.7)	6 (2.7)
	MDS	20 (4.8)	17 (7.8)
	MM	15 (3.6)	11 (5.0)
	NHL	34 (8.2)	14 (6.4)
	Others	24 (5.8)	10 (4.6)
	Total	416 (100.0)	219 (100.0)
Type of transplantation, n (%)	Homogenic	5 (2.7)	1 (0.9)
	Allogeneic	154 (81.9)	96 (88.9)
	Autologous	29 (15.4)	11 (10.2)
	Total	188 (100.0)	108 (100.0)
Posaconazole used in patients treated with chemotherapy, n (%)	No	28 (10.4)	28 (20.7)
	Yes	240 (89.6)	107 (79.3)
Type of chemotherapy, n (%)	Consolidate	116 (48.3)	54 (50.5)
	Induction	122 (50.8)	51 (47.7)
	Other	2 (0.8)	2 (1.9)
	Total	240 (100.0)	107 (100.0)

In the posaconazole prophylaxis group, 89.6% (240/268) were treated with chemotherapy, with 50.8% (122/240) receiving induction chemotherapy and 48.3% (116/240) undergoing consolidating chemotherapy. A 79.3% (107/243) patients with chemotherapy were given posaconazole as treatment, 47.7% (51/107) received induction and 50.5% (54/107) consolidation treatment (Table 1).

### 3.2 Posaconazole as prophylactic medication

The mean duration for a course of prophylactic posaconazole administration was 19.0 days, with a median of 15.0 days; the mean dose of posaconazole was 616.2 mg once a day and the median dose was 600.0 mg once a day.

#### 3.2.1 IFD breakthrough rate in patients who received prophylactic posaconazole (continuously given posaconazole for ≥4 days)

Of 445 patients who received continuous posaconazole prophylaxis for ≥4 days, probable IFDs developed in 7 cases, which led to a breakthrough rate of 1.6% (95% Cl: 0.6%–3.2%). In addition, 27 (6.1%) cases were diagnosed with possible and 29 (6.5%) with unclassified IFDs, whereas 382 (85.8%) cases were not infected with an IFD. For AML/MDS patients who received induction chemotherapy, the IFD breakthrough rate was 2.6% (2/78, 95% CI: 0.3%–9.0%). For patients who received HSCT, the IFD breakthrough rate was 2.2% (4/184, 95% CI: 0.6%–5.5%) (Table 2).

**Table 2:** The incidence of IFD and breakthrough of IFD in patients who received posaconazole as a prophylaxis drug for 4 days or more.

Index	Posaconazole as a prophylaxis drug continuously used for more than 4 days (N = 445)	Breakthrough rate (%)	95% CI
IFD final diagnostic level			
Probable IFD	7	1.6%	0.6%–3.2%
Possible IFD	27		
Unclassified IFD	29		
Non-IFD patients	382		
IFD final diagnostic level of AML/MDS patients who received induction chemotherapy			
Probable IFD	2	2.6%	0.3%–9.0%
Possible IFD	6		
Unclassified IFD	7		
Not IFD	63		
IFD final diagnostic level of patients who received HSCT			
Probable IFD	4	2.2%	0.6%–5.5%
Possible IFD	16		
Unclassified IFD	11		
Not IFD	153		

When posaconazole was used as the first-line antifungal prophylaxis medication for ≥4 days, the IFD breakthrough was 1.9% (7/372), whereas posaconazole was used as the secondary antifungal prophylaxis drug in 72 patients, with an IFD breakthrough rate of 0% (0/72). The reasons for switching medication were a consideration of the cost, side effects, intolerance, or for other reasons (Table 3).

**Table 3:** The IFD breakthrough rates and the proportion of patients who received posaconazole ≥4 days as first-line or second-line prophylaxis medication.

	First-line anti-fungal prophylaxis		Second-line anti-fungal prophylaxis	
	N	Breakthrough rate (N, %)	N	Breakthrough rate (N)
Posaconazole prophylaxis ≥4 days^∗^	372	7 (1.9)	72	0
Posaconazole as initial mono-prophylaxis	282	6 (2.1)	45	0
Posaconazole as initial combined prophylaxis medication	7	0	1	0
Other anti-fungal drug as initial prophylaxis then switched to posaconazole as second-line mono-prophylaxis	74	1 (1.4)	25	0
Other anti-fungal drug as initial prophylaxis then plus posaconazole as second-line medication	9	0	1	0

#### 3.2.2 IFD had different breakthrough rates in prophylactically medicated patients with different diseases

Of the patients administered posaconazole as first-line (n = 372) and second-line (n = 72) prophylaxis for ≥4 days, of 232 patients who received chemotherapy the IFD breakthrough rate was 1.3% (3/232) comprising only first-line prophylaxis cases since in second-line prophylaxis patients the IFD breakthrough rate was 0%.

Of a total of 109 patients who underwent consolidation chemotherapy their IFD breakthrough rate was 0.9% (1/109), whereas in patients undergoing induction chemotherapy the breakthrough rate was 1.7% (2/121).

Of the patients treated with chemotherapy, there were 151 cases of primary AML, 1 case of primary MDS, and 35 cases of primary ALL, with IFD breakthrough rates of 2.0% (3/151), 0% (0/1), and 0% (0/35), respectively (Table 4).

**Table 4:** The IFD breakthrough rates in different populations after continuous prophylaxis with posaconazole for ≥4 days.

	First-line prophylaxis		Second-line prophylaxis		Total	
Population^∗^	N	Breakthrough (N, %)	N	Breakthrough (N)	N	Breakthrough (N, %)
Chemotherapy	210	3 (1.4)	22	0	232	3 (1.3)
Transplantation	137	4 (2.9)	47	0	184	4 (2.2)
Non-chemotherapy and non-transplantation	25	0	3	0	28	0
Transplantation-allogeneic	107	3 (2.8)	43	0	150	3 (2.0)
Transplantation-syngeneic/autologous	30	1 (3.3)	4	0	34	1 (2.9)
Chemotherapy-induction	115	2 (1.7)	6	0	121	2 (1.7)
Chemotherapy-consolidation	93	1 (1.1)	16	0	109	1 (0.9)
Chemotherapy-AML	136	3 (2.2)	15	0	151	3 (2.0)
Chemotherapy-MDS	1	0	0	0	1	0
Chemotherapy-ALL	31	0	4	0	35	0

The IFD breakthrough rate in patients who received transplantation was 2.2% (4/184) and 0% in patients who did not receive chemotherapy or transplantation. For patients who received transplantation, those who had allogeneic transplantation had an IFD breakthrough rate of 2.0%, and those with syngeneic/autologous transplantation had a rate of 2.9% (Table 4). The times of prophylactic medication until the diagnosis of probable/possible IFDs for AML/MDS and HSCT patients were 11.1 ± 5.71 and 24.4 ± 18.31 days.

### 3.3 Posaconazole as a treatment for proven, probable, possible, and unclassified IFDs

The mean course of posaconazole treatment as the initial IFD medication was 22.5 days (median 14.0 days) and the mean course of treatment with posaconazole as non-initial IFD medication was 18.6 ± 19.19 days (median 13.0 days). The dosage of posaconazole for treatment was double the dose used for prophylaxis.

#### 3.3.1 The effective remission rate of ≥7-day posaconazole therapy for 243 proven/probable/possible and unclassified IFD cases

**Figure 2:**
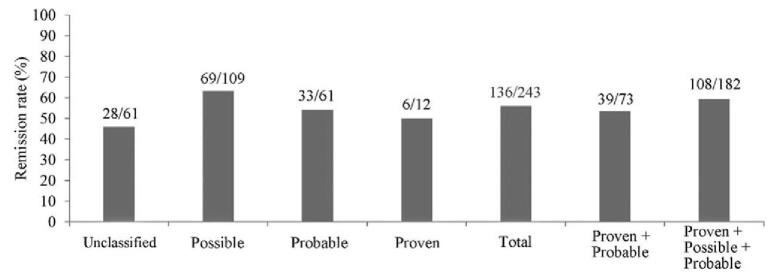
Effective remission rates of different IFD diagnosis levels at the end of posaconazole therapy for >7 days (243 cases). IFD = invasive fungal disease.

The effective remission rates of posaconazole therapy for proven, probable, possible, and unclassified in a total of 243 IFD cases after continuous administration of posaconazole for ≥7 days were 50.0% (6/12), 54.1% (33/61), 63.3% (69/109), and 45.9% (28/61), respectively. The overall effective remission rate was 56.0% (95% CI: 49.5% to 62.3%, 136/243) and that for proven, probable, possible cases was 59.3% (95% Cl: 51.8% to 66.5%, 108/182) (Fig. 2).

The effective remission rates in patients who received posaconazole monotherapy were 58.3% (95% CI: 50.0% to 66.2%, 88/151) and 52.2% (95% CI: 41.5% to 62.7%, 48/92) for patients who received combination therapy of posaconazole with other antifungal drugs. The reasons for switching medications were the same as for prophylactic drugs, that is, mainly cost, side effects, or intolerance (Table 5).

**Table 5:** Overall evaluation of posaconazole's therapeutic effect after continuous usage for ≥7 days for IFD patients at the end of treatment.

Index	Posaconazole as initial monotherapy N = 73 (%)	Posaconazole as non-initial monotherapy N = 78 (%)	Posaconazole as monotherapy N = 151 (%)	Posaconazole as initial combination therapy N = 34 (%)	Posaconazole as non-initial combination therapy N = 58 (%)	Posaconazole as combination therapy N = 92 (%)	Total N = 243 (%)
CR	9 (12.3)	5 (6.4)	14 (9.3)	7 (20.6)	5 (8.6)	12 (13.0)	26 (10.7)
PR	32 (43.8)	42 (53.8)	74 (49.0)	11 (32.4)	25 (43.1)	36 (39.1)	110 (45.3)
Unchanged	9 (12.3)	12 (15.4)	21 (13.9)	4 (11.8)	16 (27.6)	20 (21.7)	41 (16.9)
Failed	1 (1.4)	5 (6.4)	6 (4.0)	5 (14.7)	8 (13.8)	13 (14.1)	19 (7.8)
Invaluably	19 (26.0)	10 (12.8)	29 (19.2)	6 (17.6)	3 (5.2%)	9 (9.8)	38 (15.6)
Effective remission rate (95% CI)	41 (56.2) (44.1%–67.8%)	47 (60.3) (48.5%–71.2%)	88 (58.3) (50.0%–66.2%)	18 (52.9) (35.1%–70.2%)	30 (51.7) (38.2%–65.0%)	48 (52.2) 41.5%–62.7%	136 (56.0) (49.5%–62.3%)

#### 3.3.2 Effective remission rate after posaconazole as salvage therapy in proven and probable IFD patients after continuous posaconazole therapy for >7 days)

Of the 73 proven and probable IFD cases, 34 (46.6%) received posaconazole as salvage therapy. Complete and partial remission was achieved in 1 and 16 of these cases respectively, leading to an effective remission rate of 50% (95% CI: 32.4% to 67.6%; 17/34). Of the 34 patients who received continuously posaconazole as salvage therapy for >7 days, 4 received posaconazole salvage therapy due to *Aspergillus*-related IFD infections. Complete and partial remission occurred in 1 and 2 cases, respectively, leading to an effective remission rate of 75% (95% CI: 19.4% to 99.4%, 3/4) (Table 6).

**Table 6:** The clinical evaluation of effective remission rates of posaconazole salvage therapy for IFD patients.

Index	At the end of IFD treatment	Posaconazole as monotherapy (N, %)	Posaconazole as combination therapy (N, %)	Total (N, %)
Posaconazole as salvage treatment				
	CR	1 (6.7)	0	1 (2.9)
	PR	9 (60.0)	7 (36.8)	16 (47.1)
	Unchanged	2 (13.3)	7 (36.8)	9 (26.5)
	Failure	2 (13.3)	5 (26.3)	7 (20.6)
	Invaluable	1 (6.7)	0	1 (2.9)
	Total	15 (100.0)	19 (100.0)	34 (100.0)
	Effective remission rate (n, % (95% CI))	10/66.7 (38.4%–88.2%)	7 /36.8 (16.3%–61.6%)	17/50.0 (32.4%–67.6%)
Posaconazole as salvage treatment for aspergillus IFDs				
	CR	1 (100.0)	0	1 (25.0)
	PR	0	2 (66.7)	2 (50.0)
	Unchanged	0	0	0
	Failure	0	1 (33.3)	1 (25.0)
	Invaluable	0	0	0
	Total	1 (100.0)	3 (100.0)	4 (100.0)
	Effective remission rate (n, % (95% CI))	1/100.0 (2.5%–100.0%)	2/ 66.7 (9.4%–99.2%)	3/75.0 (19.4%–99.4%)

## 4 Discussion

The guidelines of the European Conference on Infections in Leukemia recommends posaconazole for primary antifungal prophylaxis of adult patients with AML and MDS undergoing remission-induction and in the pre-engraftment period of adult allogeneic HSCT recipients at high risk of contracting mold infections, as well as in the post-engraftment period of adult allogeneic HSCT recipients. Otherwise, posaconazole is the treatment of choice in centers with a high incidence (>8%) of invasive mold disease.^[21]^ Posaconazole has been in clinical use in China since early 2014. In the present study, we investigated IFD breakthrough rates after posaconazole prophylaxis or treatments in patients with hematological malignancies. In addition, we assessed the risk factors for IFD breakthrough after posaconazole prophylaxis for >4 days by univariate analysis and found chronic hepatitis (OR = 14.182 (95% CI: 2.989% to 67.296), *P* = .001) and having Epstein–Barr viremia within 2 weeks (OR = 5.933; 95% CI: 0.597% to 58.943; *P* = .038) appeared to be risk factors for IFD breakthrough, but statistical significance was not reached after subsequent multivariate analysis (*P* = .088). The total diagnosed probable IFD breakthrough rate was 1.6% (7/445) and the total probable and possible IFD breakthrough rate was 7.6% (34/445) in patients who received posaconazole continuously as prophylaxis for >4 days. This finding is clinically important because IFDs are still one of the main causes of death in hematological patients receiving high-intensity chemotherapy and/or HSCT.^[22]^ Breakthrough rates during prophylactic posaconazole therapy of 3.9% in proven or probable IFD^[16]^ and 7.5% in proven/probable/possible IFD^[18]^ have been previously reported. In a prospective study of newly diagnosed adult AML patients who received chemotherapy, the IFD breakthrough rate (probable and proven IFDs) was 2.7% (7/260) after posaconazole prophylaxis.^[23]^ An explanation for the lower IFD breakthrough rate in the present study might be that at the start of treatment, only 26% of patients were in the agranulocytosis stage (absolute neutrophil count <500/mm^3^). The probable IFD breakthrough rates in our study differed in different patients being 2.0% in AML/MDS vs 0% in ALL cases, which is in accordance with a previous review that highlighted a higher incidence of fungal infections in AML compared to ALL patients. This finding has been attributed to either an intrinsic functional defect or to a reduction of neutrophil numbers in AML cases.^[24]^

On the other hand, compared to breakthrough rates with other antifungal medications posaconazole in the present study exhibited essentially superior outcomes, since a previous Chinese study on invasive fungal infections (IFIs) in patients receiving chemotherapy or HSCTs for hematological malignancy reported that 20.4% and 35.6% of patients after primary and secondary prophylaxis treatments with triazoles, echinocandins, and amphotericin B were ultimately diagnosed with having an IFD.^[25]^ Also, another Chinese study on hematological malignancy patients receiving chemotherapy found that 11.2% of patients were mainly treated with fluconazole, itraconazole, and voriconazole as primary and secondary prophylaxes switched to alternative agents due to confirmed or suspected IFIs.^[6]^

In our study, the effective remission rates of posaconazole as monotherapy for probable and proven IFD treatments were between 56.2% and 60.3%, as well as 50.0% (95% CI: 32.4% to 95%, 17/34) after salvage treatment with posaconazole for >7 days. The rates were less than the 80% noted in a previous report from the United States,^[26]^ but similar to a study from South Korea.^[27]^ Low serum levels of posaconazole might have contributed to these IFD remission differences^[11,28]^ since strategies for improving posaconazole exposure such as administration of acidic beverages, higher doses, as well as restriction of proton pump inhibitors have been proposed.^[18]^ Treatment of *Fusariosis*, *Chromoblastomycosis*, *Coccidioidomycosis*, and *Candida* infections with posaconazole has been approved as well as treatment of invasive *Aspergillosis* infections, which are difficult to treat or show no response to deoxycholic acid AmB or itraconazole therapy, and also to alternative treatments of filamentous fungal infection (Mucor).^[29]^ These uses have been confirmed by our results with an effective remission rate of 75% for *Aspergillus* salvage therapy.

The main limitation of our investigation was its retrospective design as a single-group study to assess the clinical effects of posaconazole in a heterogeneous patient population, and that there was no control group. Unlike the strict conditions of random controlled clinical trials, there can be biases in observational studies, for example, information and selection bias. We adopted the following 2 methods to reduce the likelihood of information bias: first, we provided EORTC/MSG guidelines to each research center and all researchers for IFD diagnosis-based guidelines for case report forms; second, we performed a logical check for the diagnosis results of IFD using programming through data management to ensure that the IFD diagnosis results were in accordance with the guidelines if the objective data did not conform with the researcher's judgment. Subgroup analysis and multivariate analysis were used to reduce selection bias in clinical outcome assessments, but it is unlikely that all potential confounders were monitored; for example, it is difficult to avoid the effects of patient heterogeneity. Furthermore, the data collection in the present study was mainly based on each patient's medical history, which might have been incomplete. We focused on probable IFD breakthrough occurrence 4 days after initiation of prophylactic treatment with posaconazole and within 7 days of discontinuation of treatment. However, a small percentage of patients might have been outside the hospital and even if the researcher asked the patient by telephone if he/she had an IFD, it was still possible to miss out-of-hospital IFD cases during this period. In addition, the clinical manifestations and pathogens are not necessarily recorded in the medical record at each observation time point since it is improbable that computed tomography scans were performed at the start of posaconazole treatment, 7 days after treatment, or at the end of treatment. As different hospitals use a variety of microbiological tests, it may not have been possible to identify or record each type of fungus that had infected the enrolled patients.

In brief, the assessed overall probable IFD breakthrough rate was 1.6% after continuous prophylactic use of posaconazole for ≥4 days in 445 hematological malignancy cases, but the probable IFD breakthrough rate under prophylactic posaconazole medication was estimated to be 1.3% for patients treated with chemotherapy, 2.2% for patients who received transplantation, 2.0% for AML/MDS, and 0% for ALL patients, as well as 1.9% and 0%, respectively when using posaconazole as first-line and second-line antifungal prophylaxis therapy. The overall effective remission rate was estimated to be 56.0% in patients who received continuous posaconazole treatment for >7 days, and posaconazole monotherapy and combination therapies with other antifungal drugs had similar salvage therapy effects (58.3% and 52.2%, respectively).

## 5 Conclusions

This real-world retrospective study confirmed posaconazole as reasonable IFD prophylaxis and treatment medication for hematological patients.

## Acknowledgments

The authors would like to thank the patients and investigators at each study site, without whom this study would not have been possible. The authors accept direct responsibility for this paper and are grateful for the contribution made by Bin Chen, MD with MSD China in collating comments and providing editorial assistance.

## Author contributions

All of the authors have read and approved the manuscript. The authors were solely responsible for the conception and implementation of the study and for writing the manuscript.

**Conceptualization:** Xiaochen Chen, Jie Jin, Yu Hu, Xiaoxiong Wu, Xiaojun Huang, Jianping Shen, Depei Wu.

**Data analysis:** Xiaochen Chen, Depei Wu.

**Data collection:** Jianxiang Wang, Sanbin Wang, Jie Jin, Junmin Li, Sujun Gao, Jianyong Li, Juan Li, Qifa Liu, Yu Hu, Dongjun Lin, Zimin Sun, Jianmin Yang, Jianda Hu, Xiaoxiong Wu, Xiaojun Huang, Zonghong Shao, Qi Deng, Chun Wang, Li Liu, Hu Chen, Jingbo Wang, Xudong Wei, Jianping Shen, Xi Zhang, Depei Wu.

**Data curation:** Jianxiang Wang, Sanbin Wang, Jie Jin, Junmin Li, Sujun Gao, Jianyong Li, Juan Li, Qifa Liu, Yu Hu, Dongjun Lin, Zimin Sun, Jianmin Yang, Jianda Hu, Xiaoxiong Wu, Xiaojun Huang, Zonghong Shao, Qi Deng, Chun Wang, Li Liu, Hu Chen, Jingbo Wang, Xudong Wei, Jianping Shen, Xi Zhang, Depei Wu.

**Formal analysis:** Xiaochen Chen, Depei Wu.

**Project administration:** Depei Wu.

**Supervision:** Depei Wu.

**Validation:** Xiaochen Chen, Jianxiang Wang, Sanbin Wang, Jie Jin, Junmin Li, Sujun Gao, Jianyong Li, Juan Li, Qifa Liu, Yu Hu, Dongjun Lin, Zimin Sun, Jianmin Yang, Jianda Hu, Xiaoxiong Wu, Xiaojun Huang, Zonghong Shao, Qi Deng, Chun Wang, Li Liu, Hu Chen, Jingbo Wang, Xudong Wei, Jianping Shen, Xi Zhang, Depei Wu.

**Visualization:** Xiaochen Chen, Jianxiang Wang, Sanbin Wang, Jie Jin, Junmin Li, Sujun Gao, Jianyong Li, Juan Li, Qifa Liu, Yu Hu, Dongjun Lin, Zimin Sun, Jianmin Yang, Jianda Hu, Xiaoxiong Wu, Xiaojun Huang, Zonghong Shao, Qi Deng, Chun Wang, Li Liu, Hu Chen, Jingbo Wang, Xudong Wei, Jianping Shen, Xi Zhang, Depei Wu.

**Writing – original draft:** Xiaochen Chen, Yu Hu, Xiaoxiong Wu, Xiaojun Huang, Depei Wu.

**Writing – review & editing:** Jianxiang Wang, Sanbin Wang, Jie Jin, Junmin Li, Sujun Gao, Jianyong Li, Juan Li, Qifa Liu, Yu Hu, Dongjun Lin, Zimin Sun, Jianmin Yang, Jianda Hu, Xiaoxiong Wu, Xiaojun Huang, Zonghong Shao, Qi Deng, Chun Wang, Li Liu, Hu Chen, Jingbo Wang, Xudong Wei, Jianping Shen, Xi Zhang, Depei Wu.

## Supplementary Material

Supplemental Digital Content

## Supplementary Material

Supplemental Digital Content
