# Supplementary figures and images for: Real-world assessment of the effectiveness of posaconazole for the prophylaxis and treatment of invasive fungal infections in hematological patients: A retrospective observational study
Source: Medicine (Baltimore). 2021 Jul 30;100(30):e26772. doi: 10.1097/MD.0000000000026772 (PMC8322488; doi:10.1097/MD.0000000000026772)

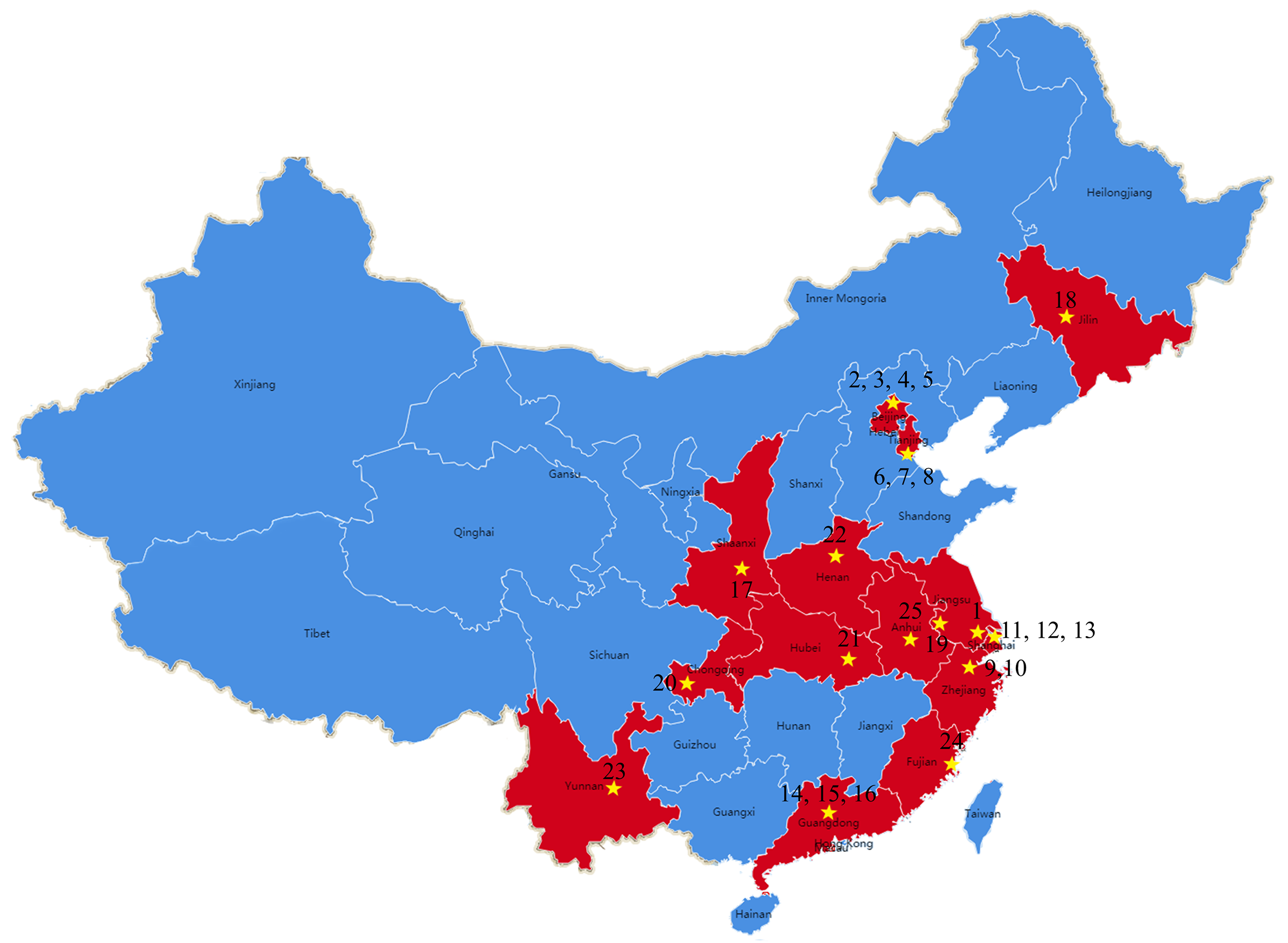

Supplement: Supplemental Digital Content [file medi-100-e26772-s001.tif]
