# Supplementary material for: Real-world assessment of the effectiveness of posaconazole for the prophylaxis and treatment of invasive fungal infections in hematological patients: A retrospective observational study
Source: Medicine (Baltimore). 2021 Jul 30;100(30):e26772. doi: 10.1097/MD.0000000000026772 (PMC8322488; doi:10.1097/MD.0000000000026772)
Supplement: Supplemental Digital Content [file medi-100-e26772-s002.docx]

**Supplementary Table 1**. Sample size calculation

| Incidence of IFD | Margin of error  with 95% CI | Sample size |
| --- | --- | --- |
| 4% | ± 2.5% | 236 |
| 3% | ± 2.5% | 179 |
| 2% | ± 2.5% | 121 |
| 1% | ± 2.5% | 61 |
